# An Extract Produced by *Bacillus* sp. BR3 Influences the Function of the GacS/GacA Two-Component System in *Pseudomonas syringae* pv. *tomato* DC3000

**DOI:** 10.3389/fmicb.2019.02005

**Published:** 2019-09-11

**Authors:** Bo Zhang, Yang Zhang, Fei Liang, Yinan Ma, Xiaogang Wu

**Affiliations:** ^1^College of Agriculture, Guangxi University, Nanning, China; ^2^Department of Plant Pathology, China Agricultural University, Beijing, China

**Keywords:** the GacS/GacA two-component system, small non-coding RNA, *Bacillus* sp., *Pseudomonas syringae* pv. *tomato*, type three secretion system

## Abstract

The GacS/GacA two-component system is essential for virulence in many plant pathogenic bacteria, and thus represents a promising anti-virulence target. In the present study, we isolated and screened rhizobacteria that were capable of inhibiting the expression of the *gacS* gene in the phytopathogenic bacterium *Pseudomonas syringae* pv. *tomato* (*Pto*) DC3000. One candidate inhibitor bacterium, BR3 was obtained and identified as a *Bacillus* sp. strain based on 16s rRNA gene sequence analysis. Besides the *gacS* gene, the GacA-dependent small RNA genes *rsmZ* and *rsmY* were repressed transcriptionally when DC3000 was treated with an extract from strain BR3. Importantly, the extract also influenced bacterial motility, the expression of type three secretion system effector AvrPto, and the plant hypersensitive response triggered by strain DC3000. The results suggested that the extract from strain BR3 might offer an alternative method to control bacterial diseases in plants by targeting the GacS/GacA system.

## Introduction

To thrive and survive in complex environmental conditions, bacteria employ versatile signal transduction pathways to rapidly adapt to alterations in their surroundings. One of these pathways is the GacS/GacA two-component system, which detects and responds coordinately to external and internal stimuli, including different physiological state (Kay et al., 2005), metabolic levels (Chavez et al., 2010), and pH (Mondragón et al., 2006), and translates them into appropriate adaptive responses.

The GacS/GacA system is a conserved global regulatory system in many Gram-negative bacteria, which is comprised of the hybrid sensor kinase GacS and the cognate response regulator GacA. In the presence of an as-yet-unidentified environmental signal, the GacS sensor kinase autophosphorylates and then activates the cognate GacA response regulator via phosphotransfer (Haas and Défago, 2005). In *Pseudomonas aeruginosa* and *Legionella pneumophila*, the activated GacA exclusively regulates the expression of several small non-coding RNAs (sRNAs) RsmY and RsmZ (Brencic et al., 2009; Sahr et al., 2009). Mutation of all these sRNAs in many bacteria resulted in the same phenotypes as mutants of the GacS/GacA system (Kay et al., 2005; Brencic et al., 2009). GacA-activated sRNAs all have multiple GGA motifs for competitive binding to carbon storage regulator (CsrA)/regulator of secondary metabolites (RsmA) family proteins (Valverde et al., 2004). In addition, the CsrA/RsmA family proteins mediate either a negative or positive posttranscriptional effect by altering the rate of translation initiation or mRNA decay (Lapouge et al., 2008). A common consensus sequence (CANGGAYG) within the loop portion of a stem–loop structure in the 5′-untranslated region (UTR) is essential for RsmA/CsrA family proteins to bind target mRNAs (Kulkarni et al., 2014). Two histidine kinases, LadS and RetS, are involved in modulating the function of the GacS/GacA system. In *P. aeruginosa* PAK, RetS affects the phosphorylation state of GacS (Goodman et al., 2009). Crystallographic studies further indicated that RetS used the reversible unfolding of a helix, or helix cracking, to control interactions with GacS (Mancl et al., 2019). In contrast to RetS, LadS in *P. aeruginosa* activates the function of GacA under high calcium conditions (Broder et al., 2016).

Extensive studies have demonstrated that the GacS/GacA system and its homologs play an important role in coordinating the expression of virulence factors required for successful infection of many plant- and animal-pathogenic bacteria (Heeb and Haas, 2001). In *P. syringae* pv. *tomato* (*Pto*) DC3000 (hereafter termed *Pto* DC3000), GacA acts as master regulator to control carbon metabolism, motility, and production of virulence factors, syringomycin, and quorum-sensing (QS) signals (Chatterjee et al., 2003). Furthermore, GacA positively regulates the transcription of the *pel*, *peh*, and *celV* genes that are responsible for the production of pectate lyases, pectinases, and cellulases in *Pectobacterium carotovorum* subsp. *carotovorum* (*Pcc*), and mutation of *gacA* results in an avirulent phenotype (Cui et al., 2001). Production of these exoenzymes was under the control of ExpI-ExpR QS system (Pirhonen et al., 1993) and the QS system was positively regulated by the GacS/GacA system (Whitehead et al., 2002). The opportunistic pathogen *P. aeruginosa* caused extensive tissue damage on *Arabidopsis* and lettuce when infiltrated at high cell densities, while the *gacA* mutant sharply reduced the disease symptoms (Parkins et al., 2001). Moreover, the *gacA* or *gacS* mutants of *P. aeruginosa* are also much less virulent in several animal models compared with their wild-type (Pessi and Haas, 2001). In addition, the GacA homologs in human pathogens *Salmonella enterica* serovar *Typhimurium* and *Vibrio cholerae* act as key regulators of colonization, toxin production, and intracellular multiplication (Wong et al., 1998; Ahmer et al., 1999).

Hence, the GacS/GacA system represents a promising target for anti-infection drug development. Although the signaling circuit is well defined, little is known about the environmental signals that turn on the Gac/Rsm regulatory cascades. Short-chain fatty acids have been shown to induce the homologous systems in *Escherichia coli* and *Salmonella typhimurium* (Lawhon et al., 2002; Gonzalez Chavez et al., 2010). Bacterial culture supernatants and lysed kin cells could act as signals that are sensed by the GacS/GacA–CsrA/RsmA pathway in *P. aeruginosa* (Kay et al., 2005; LeRous et al., 2015). In addition, plant phenolic derivatives and the antibiotic azithromycin impaired the production of virulence factors in *P. aeruginosa* via the GacS/GacA system (Pérez-Martínez and Haas, 2011; Yamazaki et al., 2012).

In the present study, a *Pto* DC3000 (p970Gm-gacSDC3000p) transcriptional fusion reporter was developed to screen inhibitors of the GacS/GacA system from secondary metabolites produced by rhizobacteria. The extract of *Bacillus* sp. BR3 significantly repressed *gacS* expression and reduced the GacS protein level, and impaired GacA-dependent expression of small RNAs, motility, and the hypersensitive response (HR) triggered by *Pto* DC3000. These results contributed to our understanding of interspecies cell-to-cell communication in bacteria, and provided an additional method by which rhizobacteria might attenuate virulence factor production by plant and animal pathogenic bacteria.

## Materials and Methods

### Bacterial Strains, Plasmids, and Growth Conditions

The bacterial strains and plasmids used in this study are listed in Table 1. *E. coli* was routinely grown in Luria-Bertani (LB) medium at 37°C. *Pseudomonas syringae* pv. *tomato* DC3000, *P. carotovorum* subsp. *carotovorum* (*Pcc*) Z3-3, *Pseudomonas fluorescens* 2P24, and *Agrobacterium tumefaciens* NTL4 (pZLR4) were cultured in LB medium, King’s Broth (KB) (King et al., 1954), or minimal medium ABM (Chilton et al., 1974) at 28°C. Type three secretion system (TTSS)-inducing minimal medium was used for immunoblotting analysis of AvrPto protein (Huynh et al., 1989). When necessary, growth media were supplemented with ampicillin (Ap) (50 μg ml^–1^), kanamycin (Km) (50 μg ml^–1^), gentamycin (Gm) (5 μg ml^–1^), tetracycline (Tet) (20 μg ml^–1^), or 5-bromo-4-chloro-3-indolyl-β-D-galactopyranoside (X-gal) (40 μg ml^–1^).

**TABLE 1:** Bacterial strains, plasmids, and primers used in this study.

**Strains or plasmids**	**Relevant characteristics**	**References or source**
**Strains**		
*Pseudomonas syringae*		
DC3000	Wild-type	Chatterjee et al., 2003
DC3000*gacA*	GacA_DC__3000_-derivative of DC3000, Km^r^	Chatterjee et al., 2003
DC3000*hrcQ-U*	DC3000 Δ*hrcQ-U*:ΩSp^r^/Sm^r^	Kvitko et al., 2007
DC3000-GacSVSV	DC3000 with a VSV-G epitope sequence tagged to the C terminus of GacS	This work
*Pseudomonas fluorescens*		
2P24	Wild-type	Yan et al., 2004
2P24**△***gacA*	Derivative of 2P24, *gacA* gene in-frame deletion, Ap^r^	Yan et al., 2004
2P24**△***retS*	Derivative of 2P24, *retS* gene in-frame deletion, Ap^r^	Liu et al., 2013
*Pectobacterium carotovorum* subsp. *carotovorum* Z3-3	Wild-type	Laboratory
*Agrobacterium tumefaciens* NTL4(pZLR4)	*A. tumefaciens* NT1 derivative carrying a *traG–lacZ* reporter fusion, AHL biosensor	Chilton et al., 1974
**Plasmids**		
p970Gm-rsmYDC3000p	Derivative of DC3000, *rsmY–lacZ* transcriptional fusion, Gm^r^	This work
p970Gm-rsmZDC3000p	Derivative of DC3000, *rsmZ–lacZ* transcriptional fusion, Gm^r^	This work
p970Gm-gacSDC3000p	Derivative of DC3000, *gacS–lacZ* transcriptional fusion, Gm^r^	This work
p970Gm-rsmYp	Derivative of 2P24, *rsmY–lacZ* transcriptional fusion, Gm^r^	Wu et al., 2012
pME6013-2P24proC	Derivative of 2P24, *proC–lacZ* translational fusion, Tet^r^	This work
pME6013-phlA	Derivative of 2P24, *phlA–lacZ* translational fusion, Tet^r^	Zhang et al., 2018
p2P24Km	Suicide plasmid with *sacB* used for homologous recombination, Km^r^	Zhang et al., 2018
p2P24Km-gacSVSV	p2P24Km with a VSV-G epitope sequence tagged to the C terminus of GacS, Km^r^	This work
pME6032	*lacI*–P*tac* expression vector, Tet^r^	Heeb et al., 2002
pME-gacS	pME6032 with *gacS* under P*tac* control	This work
Primers	Sequence (5′→ 3′)	Restriction site
rsmZp1DC3000	ATGGATCC AGTAAACCTCCCACCG	*Bam*HI
rsmZp2DC3000	ATGGATCC GCTTCGATAGTAGAGATTTAAC	*Bam*HI
RsmYBglII5p	ATGAGATCT TTTATGCGAGCAAGCTG	*Bgl*II
RsmYBglII3P	ATGAGATCT TTCACCCCGCCGTCCTGGC	*Bgl*II
gacS-promoterF	GCGGATCC TCCGTTTTAGTAGTGTGCTC	*Bam*HI
gacS-promoterR	ATGGATCC GGCACAGGGTCAGCAACAGCAC	*Bam*HI
gacS-vsv2R	TTTTCCTAATCTATTCATTTCAATATCTGTATACACACTTGTCTCCTGCATCCAG	
gacS-vsv3F	TATACAGATATTGAAATGAATAGATTAGGAAAACTCAAGAAACTGGGAATCAAAG	
gacS-vsv4R	ATGGATCC TGGCTGCCCAGCGGTGGCAG	*Bam*HI
proC-F-6013	ATGAATTC TTGCACAGCGCCTGTCCGAGCAAC	*Eco*RI
proC-R-6013	CTTGGCTGCAG AATACGCGTGTTGCTCATGACAGGTCCT	*Pst*I
gacS-*Xho*I	ATACTCGAG CTCAGCCTCGATCGTTTCCTGGGGC	*Xho*I
gacS-*Eco*RI	ATGAATTC AGAGTAATGTGCCCACACTGAC	*Eco*RI
63F	CAGGCCTAACACATGCAAGTC	
1387R	GGGCGGTGATGTACAAGGC	

*Ap, ampicillin; Gm, gentamicin; Km, kanamycin; Tet, tetracycline.*

### DNA Manipulation and PCR Amplification of 16S rRNA Gene

Chromosomal DNA from strains *Pto* DC3000 and *Bacillus* sp. BR3, plasmid DNA extraction, and other molecular assays were performed according to standard procedures (Sambrook et al., 1989). Electroporation of *Pto* DC3000 and *P. fluorescens* cells with plasmid DNA was performed as described previously (Choi et al., 2006). The oligonucleotides used are listed in Table 1.

To ascertain its taxonomic position, the genomic DNA of strain BR3 was used as a templet for PCR amplification using primers 63F and 1387R (Marchesi et al., 1998). The PCR product was sequenced by the Sanger method at Sangon Biotech Co., Ltd., Shanghai, China. The obtained sequences were compared with sequences deposited in GenBank using a BLASTN algorithm (Altschul et al., 1997).

### Construction of the Transcriptional *lacZ* Fusions and the Overexpression Plasmid

To construct the reporter strain *Pto* DC3000 (p970Gm-gacSDC3000p), the *gacS* promoter region from *Pto* DC3000 was PCR amplified using the primers gacS-promoterF/gacS-promoterR. The amplicon was then inserted into the *Bam*HI site of plasmid pRG970Gm to create the *gacS*–*lacZ* transcriptional fusion plasmid p970Gm-gacSDC3000p, which was introduced into *Pto* DC3000 via electroporation (Smith and Iglewski, 1989). In the same way, the promoter regions of *rsmY* and *rsmZ* from *Pto* DC3000 were amplified by PCR using primer pairs RsmYBglII5p/RsmYBglII3p and rsmZp1DC3000/rsmZp2DC3000, respectively. The products were then cloned into plasmid pRG970Gm to obtain p970Gm-rsmYDC3000p and p970Gm-rsmZDC3000p. These plasmids were then introduced into appropriate *Pto* strains by electroporation.

Plasmid pME-gacS expressing the *gacS* gene under the control of the isopropyl β-D-thiogalactoside (IPTG)-inducible *tac* promoter (P*tac*) was constructed as follows. The *gacS* gene was amplified by PCR with the primers gacS-*Xho*I/gacS-*Eco*RI and cloned into pME6032, yielding pME-gacS. When required, 0.25 and 0.5 mM of IPTG were added to induce the P*tac* promoter of the plasmid pME6032, respectively.

### Preparation and Screening of Extracts From Soil Bacteria

Soil samples were collected from various regions in Guangxi, China, and rhizobacteria were isolated using LB plates. The isolates were cultured in 5 ml of seed broth at 28°C for 7 days (Ling et al., 2015). One milliliter of the cultures was extracted using an equal volume of ethyl acetate. The ethyl acetate extracts were evaporated and dissolved in 200 μl of 100% dimethyl sulfoxide (DMSO). A portion (5 μl) of the extract was mixed with 1 ml of bacterial suspension of the reporter strain *Pto* DC3000 (p970Gm-gacSDC3000p). After 7 h of incubation at 28°C, the β-galactosidase activity was assayed using the Miller (1972) method.

### Swarming Assay and Hypersensitive Response Test

*Pto* DC3000 and its *gacA* mutant were cultured overnight in KB liquid medium. Then, 5 μl of the stationary phase cultures was spotted onto SWM agar medium (0.4% agar) with BR3 extract (at final a concentration of 16 or 32 μg ml^–1^). The plates were incubated overnight at 28°C and then imaged (Chatterjee et al., 2003).

The procedures for HR testing in tobacco leaves were previously published (Cui et al., 1996). Young, fully expanded third and fourth leaves from approximately 8-week-old *Nicotiana tabacum* L. cv. Samsun were used for the HR test. Bacterial cells [1 × 10^7^ colony forming units (CFU) ml^–1^] in the absence or presence of the BR3 extract were infiltrated into tobacco leaves as indicated in the figure captions. Images were acquired at 20 h after infiltration. DMSO was infiltrated as a control.

### Mutant Strain Construction and Western Blotting Analysis

To construct a C-terminal vesicular stomatitis virus glycoprotein (VSV-G) epitope-GacS fusion, a PCR-generated fragment with the sequence 5′-TATACAGATATTGAAATGAATAGATTAG-3′ was inserted in-frame before the stop codon of *gacS* and cloned into p2P24Km (Zhang et al., 2018). The resulting plasmid was introduced into strain *Pto* DC3000 by electroporation, and the wild-type copy was replaced by the modified version after two recombination events under high-sucrose stress. Substitution was confirmed by sequencing.

To analyze the effect of the BR3 extract on the protein levels of GacS and the TTSS effector AvrPto, *Pto* DC3000 and its derivatives were cultured in TTSS-inducing minimal medium at 20°C for 16 h. Cell lysates were prepared by sonication and resuspended in SDS sample buffer. The samples were separated by SDS–PAGE and protein levels were analyzed using western blotting with anti-VSV antibodies (Sangon Biotech) or anti-AvrPto antibodies (Dijk et al., 1999).

### Extraction and Detection of 2,4-Diacetylphloroglucinol and *N*-Acyl Homoserine Lactone

*Pseudomonas fluorescens* 2P24 and its derivative strains were cultured in 20 ml of KBG (KB broth supplemented with 2% glucose) as described above. The antibiotic 2,4-Diacetylphloroglucinol (DAPG) was extracted from the culture supernatant and assayed using a previously described HPLC method (Shanahan et al., 1992).

Cultures of *P. carotovorum* subsp. *carotovorum* (*Pcc*) Z3-3 were grown overnight in LB medium with DMSO or the extract of strain BR3. One milliliter of the cultures was extracted with an equal volume of ethyl acetate. The ethyl acetate extracts were dried and resuspended in 50 μl of methanol. A portion (200 μl) of *A. tumefaciens* NTL4 (pZLR4) (OD_600_ = 0.8) with 5 μl of the extract solution was incubated at 28°C for 3 h, and then β-galactosidase activities were quantified (Miller, 1972).

### Assessing the Inhibition of Pathogenicity of *Pto* DC3000 in *Arabidopsis* and Z3-3 in Chinese Cabbage by the BR3 Extract

*Arabidopsis* plants were grown in a growth room at 23°C and 70% relative humidity under a 14-h light cycle. Strain *Pto* DC3000 was grown on KB agar overnight at 28°C and resuspended in water with the extract from strain BR3 or DMSO at 10^5^ CFU μl^–1^, 20 μl of the suspension was infiltrated into 4-week-old *Arabidopsis* leaves. Bacterial populations were determined at day 0 and day 3. Three 1-cm-diameter leaf disks were collected from three independent plants, and ground in 1 ml of 10 mM MgCl_2_. Bacterial colonies were then counted 2 days after plating 10 μl from serial dilutions on KB plates. For the pathogenicity test in Chinese cabbage, *Pcc* Z3-3 was grown in LB broth at 28°C for 12 h. Cells were harvested and resuspended in phosphate-buffered saline (PBS) to 10^8^ CFU ml^–1^ with or without the BR3 extract. For each Chinese cabbage leaf, three wounds were punched and inoculated with 10 μl of bacterial suspension. Maceration symptoms were documented at 30 h post-inoculation, as described previously (Li et al., 2010).

### Statistical Analysis

All experiments were performed in triplicate. The data were analyzed and compared by performing two-sample independent *t*-tests using DPS v9.50^1^.

## Results

### The Extract of Strain BR3 Reduced the Expression of *gacS* in *Pto* DC3000

**FIGURE 1:**
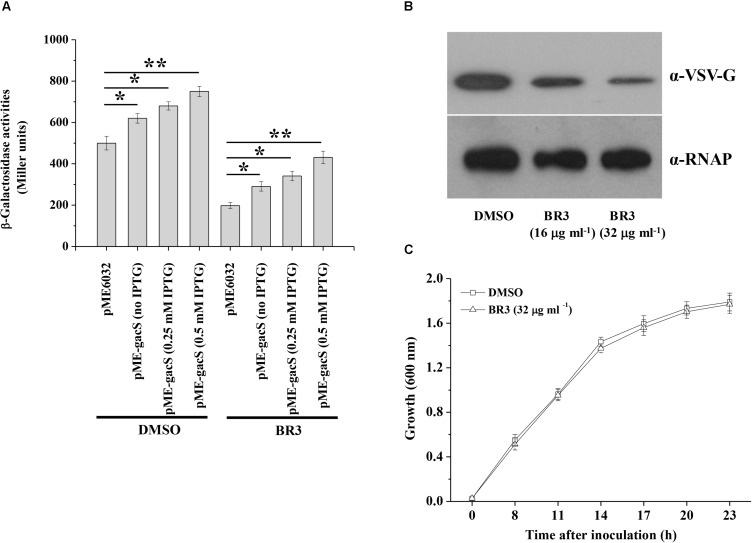
An extract of strain BR3 inhibits the expression of *gacS* and the GacS protein levels. **(A)** β-Galactosidase activities of the reporter fusion *gacS*–*lacZ* in *Pto* DC3000 (pME6032) and *Pto* DC3000 (pME-gacS) with DMSO or the BR3 extract (32 μg ml^–1^) were measured at 14 h after inoculation into KB medium. Different concentration of IPTG was added to induce the P*tac* promoter of the pME6032 as indicated. The experiments were performed in triplicate; average values ± standard deviations are shown. ^∗^*P* < 0.05 and ^∗∗^*P* < 0.01. **(B)** Western blotting analysis of GacS-VSV in the absence or presence of the BR3 extract. An antibody directed against β subunit of RNA polymerase (RNAP) was used as a loading control in this and later blots. There independent experiments were performed; a representative blot is shown. **(C)** The extract of strain BR3 (32 μg ml^–1^) did not influence the growth of *Pto* DC3000. The experiments were performed in triplicate; average values ± standard deviations are shown.

Extracts from 5000 isolates obtained from soil were tested for their abilities to suppress the β-galactosidase activity of the *gacS*–*lacZ* transcriptional fusion in the strain *Pto* DC3000 that carrying reporter plasmid p970Gm-gacSDC3000p. The extract from a strain named BR3 showed significant inhibitory activity (Figure 1A). Analysis of the 16S rRNA gene sequence of BR3 indicated that this organism belongs to *Bacillus* sp. (data not shown). Introduction of pME-gacS into the wild-type DC3000 improved the promoter activities of the *gacS* gene (Figure 1A). However, the promoter activities of *gacS* were considerably induced in the extract-treated wild-type DC3000 with plasmid pME-gacS, indicating that the expression of *gacS* gene was repressed by the BR3 extract (Figure 1A). To further verify the effect of the BR3 extract on the expression of the *gacS* gene, the level of GacS protein was determined. Western blot analysis of the chromosomal *gacS*-*vsv* fusion strain showed that GacS protein production was significantly decreased in cells treated with the BR3 extract (Figure 1B). Our data also showed that the BR3 extract had no effect on the growth of *Pto* DC3000 (Figure 1C and Supplementary Figure S1). Taken together, these results suggested that the BR3 extract suppressed the expression of *gacS* gene.

### The BR3 Extract Inhibited GacA- Dependent sRNA Expression in *Pto* DC3000

**FIGURE 2:**
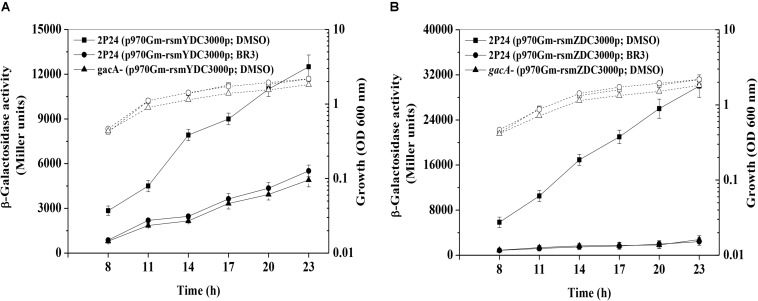
Regulation of *rsmZ* **(A)** and *rsmY* **(B)** expression in the presence or absence of the BR3 extract. β-Galactosidase activities of the reporter fusions *rsmZ*–*lacZ* **(A)** or *rsmY*–*lacZ* **(B)** in *Pto* strains with DMSO or the BR3 extract (16 μg ml^–1^) were measured at various time points tested after inoculation into KB medium. Growth is indicated by the dotted line. The experiments were performed in triplicate; average values ± standard deviations are indicated.

RsmZ-like non-coding sRNAs are regulated by the GacS/GacA system (Kay et al., 2005; Moll et al., 2010); therefore, the expression of the *rsmZ* and *rsmY* genes was further analyzed in the presence or absence of the BR3 extract. The β-galactosidase activities of the *rsmZ*–*lacZ* fusion or the *rsmY*–*lacZ* fusion in wild-type *Pto* DC3000 were significantly higher than those in *gacA* mutants, suggesting that GacA positively regulated the *rsmY* and *rsmZ* genes expression (Figure 2). Addition of the BR3 extract (16 μg ml^–1^) reduced the expression of the *rsmZ*–*lacZ* and *rsmY*–*lacZ* fusions in the wild-type *Pto* DC3000 compared with that in the DMSO-treated control (Figure 2). Interestingly, the BR3 extract had no effect on the expression of *rsmZ*–*lacZ* and *rsmY*–*lacZ* fusions in the *gacA* mutant (Figure 2). These data indicated that the BR3 extract affected the expression of *rsmZ* and *rsmY* via the GacS/GacA system.

### The Effect of the BR3 Extract on Bacterial Swarming Motility, Effector AvrPto Production, and HR on Tobacco

**FIGURE 3:**
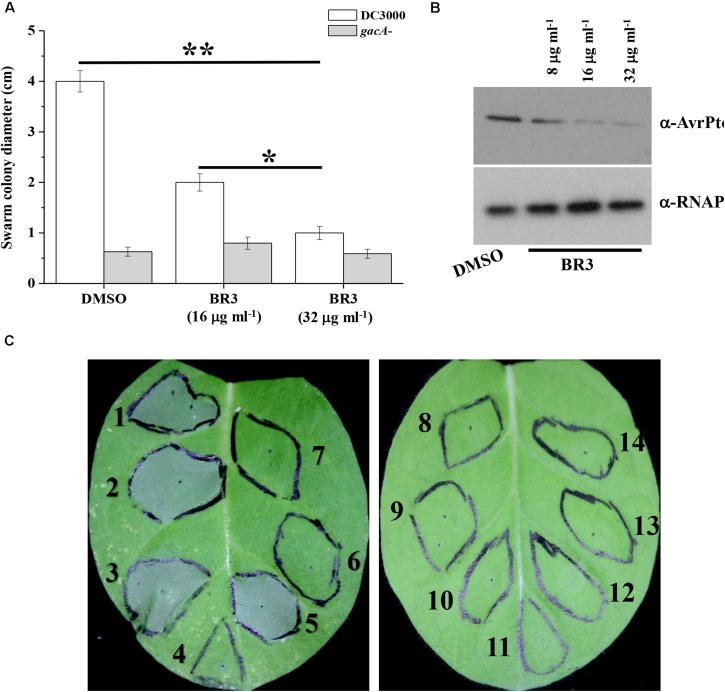
Influence of the BR3 extract on swarming motility, AvrPto production, and HR. **(A)** Swarming ability was tested on semisolid medium after 12 h of incubation at 28°C for *Pto* DC3000 in the absence or presence of the BR3 extract. Statistical significance was calculated using *t*-tests, ^∗^*P* < 0.05 and ^∗∗^*P* < 0.01. **(B)** Immunoblotting analysis with anti-AvrPto antibodies of strain *Pto* DC3000 in the absence or presence of the BR3 extract. **(C)** Effect of the BR3 extract on the elicitation of the hypersensitive response by *Pto* DC3000 in tobacco leaves. (1) DC3000 with 0 μl of DMSO; (2) DC3000 with 0 μl of the BR3 extract; (3) DC3000 with 5 μl of DMSO; (4) DC3000 with 5 μl of the BR3 extract (16 μg ml^–1^); (5) DC3000 with 10 μl of DMSO; (6) DC3000 with 10 μl of the BR3 extract (16 μg ml^–1^); (7) the *hrcQ-U* mutant with 0 μl of DMSO; (8) the *hrcQ-U* mutant with 0 μl of the BR3 extract; (9) the *hrcQ-U* mutant with 5 μl of DMSO; (10) the *hrcQ-U* mutant with 5 μl of the BR3 extract (16 μg ml^–1^); (11) the *hrcQ-U* mutant with 10 μl of DMSO; (12) the *hrcQ-U* mutant with 10 μl of the BR3 extract (16 μg ml^–1^); (13) 10 μl of DMSO; and (14) 10 μl of the BR3 extract (16 μg ml^–1^).

Previous studies indicated that the swarming motility of the *gacA* mutant was much reduced compared with that of wild-type *Pto* DC3000 (Chatterjee et al., 2003). Therefore, we checked whether the BR3 extract influenced the swarming motility of *Pto* DC3000. As expected, when treated with the extract, the wild-type displayed a swarming deficiency resembling that of its *gacA* mutant. While the swarming motility of the *gacA* mutant was not further effected upon the treatment of the BR3 extract (Figure 3A).

GacA regulates the expression of *hrpL* a master regulator of TTSS (Chatterjee et al., 2003); therefore, we further analyzed the protein level of the TTSS effector AvrPto using western blotting and observed a significant reduction in the level AvrPto in the extract-treated wild-type (Figure 3B). *Pto* DC3000 could elicit a typical HR in tobacco leaves when infiltrated at 1 × 10^7^ CFU ml^–1^ of bacterial cells, whereas the BR3 extract-treated *Pto* DC3000 failed to trigger HR at the same cell concentration (Figure 3C). In addition, the *hrcQ-U* mutant was also incapable of showing HR with or without the BR3 extract (Figure 3C). *Pto* DC3000-triggered HR on tobacco leaves is TTSS-dependent, these data suggested that the BR3 extract possible impaired the function of the TTSS in *Pto* DC3000.

### The BR3 Extract Influences *rsmY_2__P__24_* and *phlA* Expression in *P. fluorescens* 2P24

**FIGURE 4:**
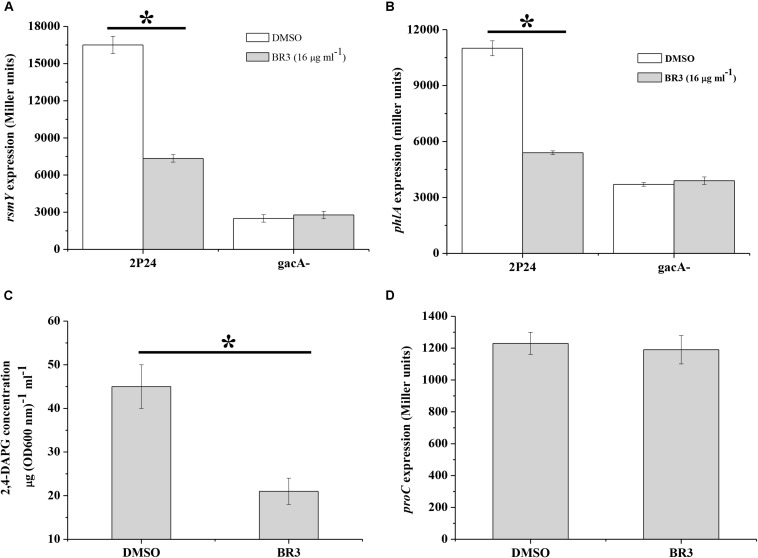
Regulation of *rsmY*, *phlA*, and *proC* of strain 2P24 in the presence or absence of the BR3 extract. The β-galactosidase activities of transcriptional *rsmY*–*lacZ* **(A)**, translational *phlA*–*lacZ* **(B)**, and *proC*–*lacZ* **(D)** fusions were determined in *P. fluorescens* 2P24 in the absence or presence of the BR3 extract. Biosynthesis of 2,4-DAPG of strain 2P24 in the absence or presence of the BR3 extract was assayed by HPLC **(C)**. All experiments were performed in triplicate, and the mean values ± standard deviations are indicated, ^∗^*P* < 0.05.

The GacS/Rsm system is conserved in many Gram-negative bacteria, and globally regulates the production of a large number of secondary metabolites, including antibiotic 2,4-DAPG in *Pseudomonas* spp. (Laville et al., 1992). We measured the influence of the BR3 extract on the expression of the sRNA gene *rsmY_2__P__24_* and 2,4-DAPG biosynthetic gene *phlA* in *P. fluorescens* 2P24. The results showed that the BR3 extract reduced *rsmY_2__P__24_* transcription and strongly inhibited the expression of the *phlA*–*lacZ* translational fusion (Figure 4). 2,4-DAPG production in the extract-treated cells reduced by approximately twofold (Figure 4C). In addition, the expression of *rsmY_2__P__24_* and *phlA* was significantly decreased in the *gacA_2__P__24_* mutant, but no further decrease was found when treated with the BR3 extract (Figures 4A,B). The expression of a housekeeping gene *proC*, which encodes the constitutive delta 1-pyrroline 5-carboxylate reductase (the third enzyme of proline biosynthesis), was not affected by the addition of the BR3 extract (Figure 4D; Savioz et al., 1993). In conclusion, our data suggested that the BR3 extract strongly inhibited the genes of the GacS/GacA regulon in *P. fluorescens* 2P24, and this inhibitory effect is GacA-dependent.

### Effect of the BR3 Extract on the Virulence of *Pto* DC3000 and *Pectobacterium carotovorum* subsp. *carotovorum* Z3-3

**FIGURE 5:**
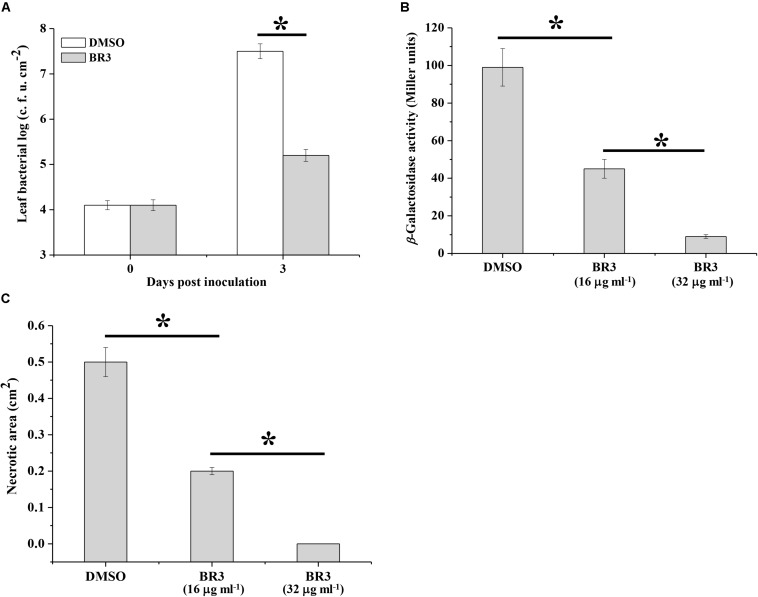
Effect of the extract of strain BR3 on the virulence function of *Pto* DC3000 and *Pcc* Z3-3. **(A)** Strain DC3000 in the absence or presence of the extract (16 μg ml^–1^) from strain BR3 was infiltrated into *Arabidopsis* leaves, and bacterial growth was determined. **(B)** Regulation by the BR3 extract on the production of AHL in *Pcc* Z3-3. The β-galactosidase activities of the *traG*–*lacZ* fusion in *A. tumefaciens* NTL4 (pZLR4) were measured via AHL signals that were extracted from strain Z3-3 in the absence or presence of the extract from strain BR3. **(C)** Disease lesions on Chinese cabbage caused by strain Z3-3 in the absence or presence of the BR3 extract. All experiments were performed in triplicate, and the mean values ± standard deviations are indicated, ^∗^*P* < 0.05.

In phytopathogenic bacteria, the Gac/Rsm system is associated with pathogenicity. The observation that the BR3 extract suppressed the activity of the Gac/Rsm system prompted us to determine whether it interfered with the GacS/GacA system-dependent virulence process. As predicted, the extract-treated *Pto* DC3000 displayed a significantly reduced *in planta* population compared with that of the *Pto* DC3000 strain treated with DMSO (Figure 5A).

Previous data indicated that the GacS/GacA system positively regulates the ExpI/ExpR QS system, which participates in the regulatory pathway for exoenzyme production in *Pcc* (Cui et al., 2001; Whitehead et al., 2002). Therefore, we further examined the influence of the BR3 extract on the pathogenicity of *Pcc* toward Chinese cabbage. The BR3 extract significantly inhibited QS signal molecules [*N*-acyl homoserine lactone (AHL)] production and the virulence of *Pcc* on Chinese cabbage (Figures 5B,C). Thus, the GacS/GacA system antagonist present in the BR3 extract is capable of disrupting GacS/GacA system-directed pathogenicity in phytopathogenic bacteria.

## Discussion

The interaction between pathogens and plants plays a critical role in plant development and fitness, including cellular mechanisms, growth, reproduction, hormonal signaling, and tolerance to environmental stresses are involved in this process (Saleem et al., 2017a). Furthermore, the interactions of bacterial species in the phyllosphere influence plant development and fitness (Saleem et al., 2017b). It is challenging to elucidate the detailed mechanisms of the effects of the microbiota on plant function (Haas and Défago, 2005).

Many plant growth-promoting rhizobacteria suppress plant disease by producing one or several antibiotic compounds to inhibit the growth or directly kill the pathogens (Haas and Défago, 2005). However, the resistance of pathogens developed quickly under the antibiotic stress and is becoming a potential challenge for the biological control of soil-borne pathogens (Spellberg and Shales, 2014; Deising et al., 2017). Other bacterial virulence-related factors, such as two-component systems, QS systems, disulfide bond forming enzymes, and TTSSs, have been considered as attractive targets of the chemical therapy (Cegelski et al., 2008; Landeta et al., 2015). The GacS/GacA system was one of these targets because of its global role in regulation of bacterial pathogenesis (Heeb and Haas, 2001).

In the present study, we developed a phytopathogenic reporter bacterium *Pto* DC3000 (p970Gm-gacSDC3000p) to screen the GacS/GacA system inhibitors from extracts of rhizobacteria. Our data indicated that the extract of *Bacillus* sp. BR3 inhibited the expression of *gacS* and its downstream genes *rsmY* and *rsmZ*, and the influence of *rsmY* and *rsmZ* expression is GacA-dependent, indicating the GacS–GacA system as the potential target of the BR3 extract (Figures 1, 2). In contrast, a previous genetic study of the human opportunistic pathogen *P. aeruginosa* suggested that the antibiotic azithromycin impaired the expression of *rsmY* and *rsmZ* regardless of the function of the GacS/GacA system, because azithromycin could reduce the expression of *rsmY* and *rsmZ* in the *gacA* mutant. Azithromycin might exert its function on genetic factors other than the GacS/GacA system, which then regulate the expression of *rsmZ* (Humair et al., 2010).

Although the GacS/GacA system plays a critical role in the production of extracellular factors, ecological fitness, and even primary metabolism (Heeb and Haas, 2001; Takeuchi et al., 2012), the chemical signals that stimulate the GacS/GacA system remain obscure. Dubuis and Haas (2007) found that extracts from culture supernatants of *Pseudomonas* sp. and *Vibrio* sp. induced the expression of sRNAs and the production of antibiotic compounds, indicating that the signals could be self-produced. Our study further demonstrated that the BR3 extract could interfere with multiple targets of the GacS/GacA regulon in different bacterial species. In addition, overexpression of the *gacS* gene could attenuate the inhibition effect of the extract BR3 on the promoter activities of *gacS* (Figure 1A). These data suggested that the BR3 extract inhibits the function of the GacS/GacA system by modulating the expression of *gacS* gene. To date, few regulatory elements have been identified to influence the expression of *gacS* (Hrabak and Willis, 1992). Our data showed that the BR3 extract could influence the GacS-dependent phenotypes in strains DC3000, 2P24, and Z3-3, whereas no conserved motif was found in the promoter regions of the *gacS* gene (data not shown), suggesting that the effect of the BR3 extract on *gacS* transcription may involve a potential regulator.

Although the method for isolation of the BR3 extract was similar to that of the AHL, the activity of the BR3 extract was not affected at pH 12 and 30°C for 1 h (data not shown). Under the same conditions, AHLs are be degraded (Swift et al., 2001). Moreover, the typical reporter system *A. tumefaciens* with the *traG*–*lacZ* fusion failed to show any reaction with the BR3 extract (Supplementary Figure S2A). These data suggested that the active ingredient from the BR3 extract does not belong to the AHL signals. Isolation of active ingredient from the BR3 extract showed that a pure compound named CX03 (MW = 413.2659) has the same inhibitory activity with the BR3 extract (Supplementary Figures S2B,C). Plant immunity is modulated by complex regulatory networks in response to abiotic and biotic factors (Jones and Dangl, 2006). Interestingly, our data showed that HR was restricted by the BR3 extract (Figure 3C). However, the BR3 extract had no effect on the transcript level of *PR1* (At2g14610) in *Arabidopsis* (data not shown). Further studies are needed to solve the chemical formula of compound CX03 and to investigate the mechanism of CX03 on plant immunity.

Rhizobacteria deploy several strategies, such as the production of antibiotics, QS, and type six secretion systems, to disrupt or otherwise manipulate commensal bacteria to facilitate their colonization and survival (Quiñones et al., 2004; Vacheron et al., 2019). Our data explored the possibility that some bacteria produce specific secondary metabolites to influence the signal transduction of others. *Bacillus* species are ubiquitous in the soil environment, and some of them are beneficial to plant growth or suppression of plant diseases (Saleem et al., 2017b); therefore, our findings deepen our understanding of the biocontrol mechanisms used by root-associated *Bacillus* spp.

Collectively, the extract from strain BR3 caused dysfunction of the GacS/GacA system, leading to attenuated activity of virulence factors, thus it may form the basis of a new anti-virulence strategy.

## Data Availability

The datasets generated for this study can be found in NCBI accession MK864268, https://www.ncbi.nlm.nih.gov/nuccore/MK864268.

## Author Contributions

BZ, YZ, FL, and XW conceived and designed the experiments. BZ, YZ, FL, and YM conduced most of the experiments. XW wrote the manuscript and all authors reviewed the manuscript critically.

## Conflict of Interest Statement

The authors declare that the research was conducted in the absence of any commercial or financial relationships that could be construed as a potential conflict of interest.

## Footnotes

1 http://www.dpsw.cn/dps_eng/index.html

## Supplementary Material

FIGURE S1 *Bacillus* sp. BR3 antagonism assays on agar plates. Strain DC3000 **(A)**, Z3-3 **(B)**, or 2P24 **(C)** were grown in LB at 28°C overnight and mixed with LB agar (ca. 50°C) to pour into the Petri dish, respectively. Five microliters of BR3 culture (1), the extract BR3 (32 μg ml^–1^) (2), and kanamycin (50 μg ml^–1^) (3) were spotted on the plates. The plates were inoculated at 28°C for 36 h. There independent experiments were performed; a representative result is shown.

FIGURE S2 **(A)** The *traG*-*lacZ* fusion in *A. tumefaciens* was not induced by the BR3 extract. β-Galactosidase activities of a *traG*-*lacZ* fusion were determined in AHL reporter strain NLT4 with the BR3 extract (32 μg ml^–1^), and the 3-oxo-hexanoyl-homoserine lactone (3-oxo-C8-HSL) (3 μg ml^–1^). **(B)** Regulation of *rsmY* expression in the presence of the BR3 extract and CX03. β-Galactosidase activities of the reporter fusion *rsmY*-*lacZ* in strain DC3000 with DMSO, the BR3 extract (16 μg ml^–1^), or CX03 (2 μg ml^–1^) were measured. **(C)** Isolation of active ingredient from the BR3 extract using HPLC and LC–MS.

## References

[B1] Ahmer B. M. Reeuwijk J. V. Watson P. R. Wallis T. S. Heffron F. (1999). *Salmonella* SirA is a global regulator of genes mediating enteropathogenesis. *Mol. Microbiol.* 31 971–982. 10.1046/j.1365-2958.1999.01244.x 10048039

[B2] Altschul S. F. Madden T. L. Schaffer A. A. Zhang J. Zhang Z. Miller W. (1997). Gapped BLAST and PSI-BLAST: a new generation of protein database search programs. *Nucleic Acids Res.* 25 3389–3402. 10.1093/nar/25.17.3389 9254694 PMC146917

[B3] Brencic A. McFarland K. A. McManus H. R. Castang S. Mogno I. Dove S. L. (2009). The GacS/GacA signal transduction system of *Pseudomonas aeruginosa* exclusively through its control over the transcription of the RsmY and RsmZ regulatory small RNAs. *Mol. Microbiol.* 73 434–445. 10.1111/j.1365-2958.2009.06782.x 19602144 PMC2761719

[B4] Broder U. N. Jaeger T. Jenal U. (2016). LadS is a calcium-responsive kinase that induces acute-to-chronic virulence switch in *Pseudomonas aeruginosa*. *Nat. Microbiol.* 2:16184. 10.1038/nmicrobiol.2016.184 27775685

[B5] Cegelski L. Marshall G. R. Eldridge G. R. Hultgren S. J. (2008). The biology and future prospects of antivirulence therapies. *Nat. Rev. Microbiol.* 6 17–27. 10.1038/nrmicro1818 18079741 PMC2211378

[B6] Chatterjee A. Cui Y. Yang H. Collmer A. Alfano J. R. Chatterjee A. K. (2003). GacA, the response regulator of a two-component system, acts as a master regulator in *Pseudomonas syringae* pv. tomato DC3000 by controlling regulatory RNA, transcriptional activators, and alternate sigma factors. *Mol. Plant Microbe Interact.* 16 1106–1117. 10.1094/MPMI.2003.16.12.1106 14651344

[B7] Chavez R. G. Alvarez A. F. Romeo T. Georgellis D. (2010). The physiological stimulus for the BarA sensor kinase. *J. Bacteriol.* 192 2009–2012. 10.1128/JB.01685-9 20118252 PMC2838055

[B8] Chilton M. D. Currier T. C. Farrand S. K. Bendich A. J. Gordon M. P. Nester E. W. (1974). *Agrobacterium tumefaciens* DNA and PS8 bacteriophage DNA not detected in crown gall tumors. *Proc. Natl. Acad. Sci. U.S.A.* 71 3672–3676. 10.1073/pnas.71.9.3672 4530328 PMC433838

[B9] Choi K. H. Kumar A. Schweizer H. P. (2006). A 10-min method for preparation of highly electrocompetent *Pseudomonas aeruginosa* cells: application for DNA fragment transfer between chromosomes and plasmid transformation. *J. Microbiol. Methods.* 64 391–397. 10.1016/j.mimet.2005.06.001 15987659

[B10] Cui Y. Chatterjee A. Chatterjee A. K. (2001). Effects of the two-component system comprising GacA and GacS of *Erwinia carotovora* subsp. carotovora on the production of global regulatory rsmB RNA, extracellular enzymes, and HarpinEcc. *Mol. Plant Microbe Interact.* 14 516–526. 10.1094/MPMI.2001.14.4.516 11310739

[B11] Cui Y. Madi L. Mukherjee A. Dumenyo C. K. Chatterjee A. K. (1996). The RsmA- mutants of *Erwinia carotovora* subsp. carotovora strain *Ecc*71 overexpress *hrpNEcc* and elicit a hypersensitive reaction-like response in tobacco leaves. *Mol. Plant Microbe Interact.* 9 565–573. 8810071 10.1094/mpmi-9-0565

[B12] Deising H. B. Gase I. Kubo Y. (2017). The unpredictable risk imposed by microbial secondary metabolites: how safe is biological control of plant diseases? *J. Plant Dis. Prot.* 124 413–419. 10.1007/s41348-017-0109-5

[B13] Dijk K. V. Fouts D. E. Rehm A. H. Hill A. R. Collmer A. Alfano J. R. (1999). The Avr (Effector) proteins HrmA (HopPsyA) and AvrPto are secreted in culture from *Pseudomonas syringae* pathovars via the Hrp (Type III) protein secretion system in a temperature- and pH-sensitive manner. *J. Bacteriol.* 181 4790–4797. 10438746 10.1128/jb.181.16.4790-4797.1999 PMC93963

[B14] Dubuis C. Haas D. (2007). Cross-species GacA-controlled induction of antibiosis in *Pseudomonads*. *Appl. Environ. Microbiol.* 73 650–654. 10.1128/AEM.01681-06 17098922 PMC1796963

[B15] Gonzalez Chavez R. Alvarez A. F. Romeo T. Georgellis D. (2010). The physiological stimulus for the *barA* sensor kinase. *J. Bacterial.* 192 2009–2012. 10.1128/JB.01685-09 20118252 PMC2838055

[B16] Goodman A. L. Merighi M. Hyodo M. Ventre I. Filloux A. Lory S. (2009). Direct interaction between sensor kinase proteins mediates acute and chronic disease phenotypes in a bacterial pathogen. *Genes Dev.* 23 249–259. 10.1101/gad.1739009 19171785 PMC2648536

[B17] Haas D. Défago G. (2005). Biological control of soil-borne pathogens by fluorescent pseudomonads. *Nature Rev. Microbiol.* 3 307–319. 10.1038/nrmicro1129 15759041

[B18] Heeb S. Blumer C. Haas D. (2002). Regulatory RNA as mediator in GacA/RsmA-dependent global control of exoproduct formation in *Pseudomonas fluorescens* CHA0. *J Bacteriol.* 184 1046–1056. 10.1128/jb.184.4.1046-1056.2002 11807065 PMC134805

[B19] Heeb S. Haas D. (2001). Regulatory roles of the GacS/GacA two-component system in plant-associated and other Gram-Negative bacteria. *Mol. Plant Microbe Interact.* 14 1351–1363. 10.1094/MPMI.2001.14.12.1351 11768529

[B20] Hrabak E. M. Willis D. K. (1992). The *lemA* gene required for pathogenicity of *Pseudomonas syringae* pv. syringae on bean is a member of a family of two-component regulators. *J. Bacteriol.* 174 3011–3020. 10.1128/jb.174.9.3011-3020.1992 1314807 PMC205956

[B21] Humair B. Wackwitz B. Haas D. (2010). GacA-controlled activation of promoters for small RNA genes in *Pseudomonas fluorescens*. *Appl. Environ. Microbiol.* 76 1497–1506. 10.1128/AEM.02014-09 20048056 PMC2832403

[B22] Huynh T. V. Dahlbeck D. Staskawicz B. J. (1989). Bacterial blight of soybean: regulation of a pathogen gene determining host cultivar specificity. *Science* 245 1374–1377. 10.1126/science.2781284 2781284

[B23] Jones J. D. Dangl J. L. (2006). The plant immune system. *Nature* 444 323–329. 10.1038/nature05286 17108957

[B24] Kay E. Dubuis C. Haas D. (2005). Three small RNAs jointly ensure secondary metabolism and biocontrol in *Pseudomonas fluorescens* CHA0. *Proc. Natl. Acad. Sci. U.S.A.* 102 17136–17141. 10.1073/pnas.0505673102 16286659 PMC1287983

[B25] King E. O. Ward M. K. Raney D. E. (1954). Two simple media for the demonstration of pyocyanin and fluorescein. *J. Lab. Clin. Med.* 44 301–307. 13184240

[B26] Kulkarni P. R. Jia T. Kuehne S. A. Kerkering T. M. Morris E. R. Searle M. S. (2014). A sequence-based approach for prediction of CsrA/RsmA targets in bacteria with experimental validation in *Pseudomonas aeruginosa*. *Nucleic Acids Res.* 42 6811–6825. 10.1093/nar/gku309 24782516 PMC4066749

[B27] Kvitko B. H. Ramos A. R. Morello J. E. Oh H.-S. Collmer A. (2007). Identification of harpins in *Pseudomonas syringae* pv. tomato DC3000, which are functionally similar to HrpK1 in promoting translocation of type III secretion system effectors. *J. Bacteriol.* 189 8059–8072. 10.1128/JB.01146-07 17873033 PMC2168707

[B28] Landeta C. Blazyk J. L. Hatahet F. Meehan B. M. Eser M. Myrick A. (2015). Compounds targeting disulfide bond forming enzyme DsbB of Gram-negative bacteria. *Nature Chem. Biol.* 11 292–298. 10.1038/NCHEMBIO.1752 25686372 PMC4366281

[B29] Lapouge K. Schubert M. Allain F. H. Haas D. (2008). Gac/Rsm signal transduction pathway of gamma-*proteobacteria*: from RNA recognition to regulation of social behaviour. *Mol. Microbiol.* 67 241–253. 10.1111/j.1365-2958.2007.06042.x 18047567

[B30] Laville J. Voisard C. Keel C. Maurhofer M. Défago G. Haas D. (1992). Global control in *Pseudomonas fluorescens* mediating antibiotic synthesis and suppression of black root rot of tobacco. *Proc. Natl. Acad. Sci. U.S.A.* 89 1562–1566. 10.1073/pnas.89.5.1562 1311842 PMC48492

[B31] Lawhon S. D. Maurer R. Suyemoto M. Altier C. (2002). Intestinal short-chain fatty acids alter *Salmonella typhimurium* invasion gene expression and virulence through BarA/SirA. *Mol. Microbiol.* 46 1451–1464. 10.1046/j.1365-2958.2002.03268.x 12453229

[B32] LeRous M. Kirkpatrick R. L. Montauti E. I. Tran B. Q. Peterson S. B. Harding B. N. (2015). Kin cell lysis is a danger signal that activates antibacterial pathways of *Pseudomonas aeruginosa*. *eLife* 4:e05701. 10.7554/eLife.05701 25643398 PMC4348357

[B33] Li Y. Yamazaki A. Zou L. Biddle E. Zeng Q. Wang Y. (2010). ClpXP protease regulates the Type III secretion system of *Dickeya dadantii* 3937 and is essential for the bacterial virulence. *Mol. Plant Microbe Interact.* 23 871–878. 10.1094/MPMI-23-7-0871 20521950

[B34] Ling L. L. Schneider T. Peoples A. J. Spoering A. L. Engels I. Conlon B. P. (2015). A new antibiotic kills pathogens without detectable resistance. *Nature* 517 455–459. 10.1038/nature14303 25561178 PMC7414797

[B35] Liu J. Zhang W. Wu X. Zhang L. (2013). Effect of *retS* gene on biosynthesis of 2,4-diacetylphloroglucinol in *Pseudomonas fluorescens* 2P24. *Acta Microbiol. Sin.* 53 118–126. 23627104

[B36] Mancl J. M. Ray W. K. Helm R. F. Schubot F. D. (2019). Helix cracking regulates the critical interaction between RetS and GacS in *Pseudomonas aeruginosa*. *Structure* 27 785–793. 10.1016/j.str.2019.02.006 30879888

[B37] Marchesi J. R. Sato T. Weightman A. J. Martin T. A. Fry J. C. Hiom S. J. (1998). Design and evaluation of useful bacterium-specific PCR primers that amplify genes coding for bacterial 16S rDNA. *Appl. Environ. Microbiol.* 64 795–799. 9464425 10.1128/aem.64.2.795-799.1998 PMC106123

[B38] Miller J. H. (1972). *Experiments in Molecular Genetics.* New York, NY: Cold Spring Harbor Laboratory.

[B39] Moll S. Scheider D. J. Stodghill P. Myers C. R. Cartinhour S. W. Filiatrault M. J. (2010). Construction of an *rsmX* co-variance model and identification of five *rsmX* non-coding RNAs in *Pseudomonas syrigae* pv. tomato DC3000. *RNA Biol*. 7 508–516. 10.4161/rna.7.512687 21060253 PMC3073247

[B40] Mondragón V. Franco B. Jonas K. Suzuki K. Romeo T. Melefors Ö, et al. (2006). pH-Dependent activation of the BarA-UvrY two-component system in *Escherichia coli*. *J. Bacteriol.* 188 8303–8306. 10.1128/JB.01052-6 16980446 PMC1698187

[B41] Parkins M. D. Ceri H. Storey D. G. (2001). *Pseudomonas aeruginosa* GacA, a factor in multihost virulence, is also essential for biofilm formation. *Mol. Microbiol.* 40 1215–1226. 10.1046/j.1365-2958.2001.02469.x 11401724

[B42] Pérez-Martínez I. Haas D. (2011). Azithromycin inhibits expression of the GacA-dependent small RNAs RsmY and RsmZ in *Pseudomonas aeruginosa*. *Appl. Environ. Microbiol.* 55 3399–3405. 10.1128/AAC.01801-10 21537014 PMC3122407

[B43] Pessi G. Haas D. (2001). Dual control of hydrogen cyanide biosynthesis by the global activator GacA in *Pseudomonas aeruginosa* PAO1. *FEMS Microbiol. Lett.* 200 73–78. 10.1111/j.1574-6968.2001.tb10695.x 11410352

[B44] Pirhonen M. Flego D. Heikinheimo R. Palva E. T. (1993). A small diffusible signal molecule is responsible for the global control of virulence and exoenzyme production in the plant pathogen *Erwinia carotovora*. *EMBO J.* 12 2467–2476. 10.1002/j.1460-2075.1993.tb05901.x 8508772 PMC413482

[B45] Quiñones B. Pujol C. J. Lindow S. E. (2004). Regulation of AHL production and its contribution to epiphytic fitness in *Pseudomonas syringae*. *Mol. Plant Microbe Interact.* 17 521–531. 10.1094/MPMI.2004.17.5.521 15141956

[B46] Sahr T. Bruggemann H. Jules M. Lomma M. Albert-Weissenberger C. Cazalet C. (2009). Two small ncRNAs jointly govern virulence and transmission in *Legionella pneumophila*. *Mol. Microbiol.* 72 741–762. 10.1111/j.1365-2958.2009.06677.x 19400772 PMC2888818

[B47] Saleem M. Ji H. Amirullah A. A. Traw M. B. (2017a). *Pseudomonas syringae* pv. tomato DC3000 growth in multiple gene knockouts predicts interactions among hormonal, biotic and abiotic stress responses. *Eur. J. Plant Pathol*. 149 779–786. 10.1007/s10658-017-1223-8

[B48] Saleem M. Meckes N. Pervaiz Z. H. Traw M. B. (2017b). Microbial interactions in the phyllosphere increase plant performance under herbivore biotic stress. *Front. Microbiol.* 8:41. 10.3389/fmicb.2017.00041 28163703 PMC5247453

[B49] Sambrook J. Fritsch E. F. Maniatis T. (1989). *Molecular Cloning: A Laboratory Manual*, 2nd Edn New York NY: Cold Spring Harbor Laboratory Press.

[B50] Savioz A. Zimmermann A. Haas D. (1993). *Pseudomonas aeruginosa* promoters which contain a conserved GG-N10-GC motif but appear to be RpoN-independent. *Mol. Gen. Genet.* 238 74–80. 8479442 10.1007/BF00279533

[B51] Shanahan P. O’Sullivan D. J. Simpson P. Glennon J. D. O’Gara F. (1992). Isolation of 2,4-diacetylphloroglucinol from a fluorescent pseudomonad and investigation of physiological parameters influencing its production. *Appl. Environ. Microbiol.* 58 353–358. 16348633 10.1128/aem.58.1.353-358.1992 PMC195214

[B52] Smith A. W. Iglewski B. H. (1989). Transformation of *Pseudomonas aeruginosa* by electroporation. *Nucleic Acids Res.* 17:10509. 10.1093/nar/17.24.10509 PMC335334 2513561

[B53] Spellberg B. Shales D. (2014). Prioritized current unmet needs for antibacterial therapies. *Clin. Pharmacol. Ther.* 96 151–153. 10.1038/clpt.2014.106 25056396

[B54] Swift S. Downie J. A. Whitehead N. A. Barnard M. L. Salmond G. P. C. Williams P. (2001). Quorum sensing as a population-density-dependent determinant of bacterial physiology. *Adv. Microb. Physiol.* 45 199–270. 10.1016/S0065-2911(01)45005-3 11450110

[B55] Takeuchi K. Yamada K. Haas D. (2012). ppGpp controlled by the Gac/Rsm regulatory pathway sustains biocontrol activity in *Pseudomonas fluorescens* CHA0. *Mol. Plant Microbe Interact.* 25 1440–1449. 10.1094/MPMI-02-12-0034-R 23035953

[B56] Vacheron J. Péchy-Tarr M. Brochet S. Heiman C. M. Stojiljkovic M. Maurhofer M. (2019). T6SS contributes to gut microbiome invasion and killing of an herbivorous pest insect by plant-beneficial *Pseudomonas protegens*. *ISME J.* 13 1318–1329. 10.1038/s41396-019-0353-8 30683920 PMC6474223

[B57] Valverde C. Lindell M. Wagner E. G. H. Haas D. (2004). A repeated GGA motif is critical for the activity and stability of the riboregulatory RsmY of *Pseudomonas fluorescens*. *J. Biol. Chem.* 279 25066–25074. 10.1074/jbc.M401870200 15031281

[B58] Whitehead N. A. Byers J. T. Commander P. Corbett M. J. Coulthurst S. J. Everson L. (2002). The regulation of virulence in phytophathogenic *Erwinia* species: quorum sensing, antibiotics and ecological considerations. *Antonie Van Leeuwenhoek* 81 223–231. 12448721 10.1023/a:1020570802717

[B59] Wong S. M. Carroll P. A. Rahme L. G. Ausubel F. M. Calderwood S. B. (1998). Modulation of expression of the ToxR regulon in *Vibrio cholerae* by a member of the two-component family of response regulators. *Infect. Immun.* 66 5854–5861. 9826365 10.1128/iai.66.12.5854-5861.1998 PMC108741

[B60] Wu X. Liu J. Zhang W. Zhang L. (2012). Multiple-level regulation of 2,4-diacetylphloroglucinol production by the sigma regulator PsrA in *Pseudomonas fluorescens* 2P24. *PLoS One* 7:e50149. 10.1371/journal.pone.0050149 23209661 PMC3510223

[B61] Yamazaki A. Li J. Zeng Q. Khokhani D. Hutchins W. C. Yost A. C. (2012). Derivatives of plant phenolic compound affect the type III secretion system of *Pseudomonas aeruginosa* via a GacS-GacA two-component signal transduction system. *Antimicrob. Agents Chemother.* 56 36–43. 10.1128/AAC.00732-11 21968370 PMC3256035

[B62] Yan X. Zhang L. Yang Z. Tang W. (2004). The role of regulatory gene *gacA* in the suppress ion of soil-borne diseases by *Pseudomonas fluorescens* 2P24. *Acta Phyto. Pathol. Sin.* 34 272–279.

[B63] Zhang Y. Zhang Y. Zhang B. Wu X. Zhang L. (2018). Effect of carbon sources on production of 2,4-diacetylphloroglucinol in Pseudomonas fluorescens 2P24. *Acta Microbiol. Sin.* 58 1202–1212. 10.13343/j.cnki.wsxb.20170356

