# Supplementary material for: An Extract Produced by Bacillus sp. BR3 Influences the Function of the GacS/GacA Two-Component System in Pseudomonas syringae pv. tomato DC3000
Source: Front Microbiol. 2019 Sep 11;10:2005. doi: 10.3389/fmicb.2019.02005 (PMC6749012; doi:10.3389/fmicb.2019.02005)
Supplement: FIGURE S1 — Bacillus sp. BR3 antagonism assays on agar plates. Strain DC3000 (A), Z3-3 (B), or 2P24 (C) were grown in LB at 28°C overnight and mixed with LB agar (ca. 50°C) to pour into the Petri dish, respectively. Five microliters of BR3 culture (1), the extract BR3 (32 μg ml–1) (2), and kanamycin (50 μg ml–1) (3) were spotted on the plates. The plates were inoculated at 28°C for 36 h. There independent experiments were performed; a representative result is shown. [file Table_1.DOC]

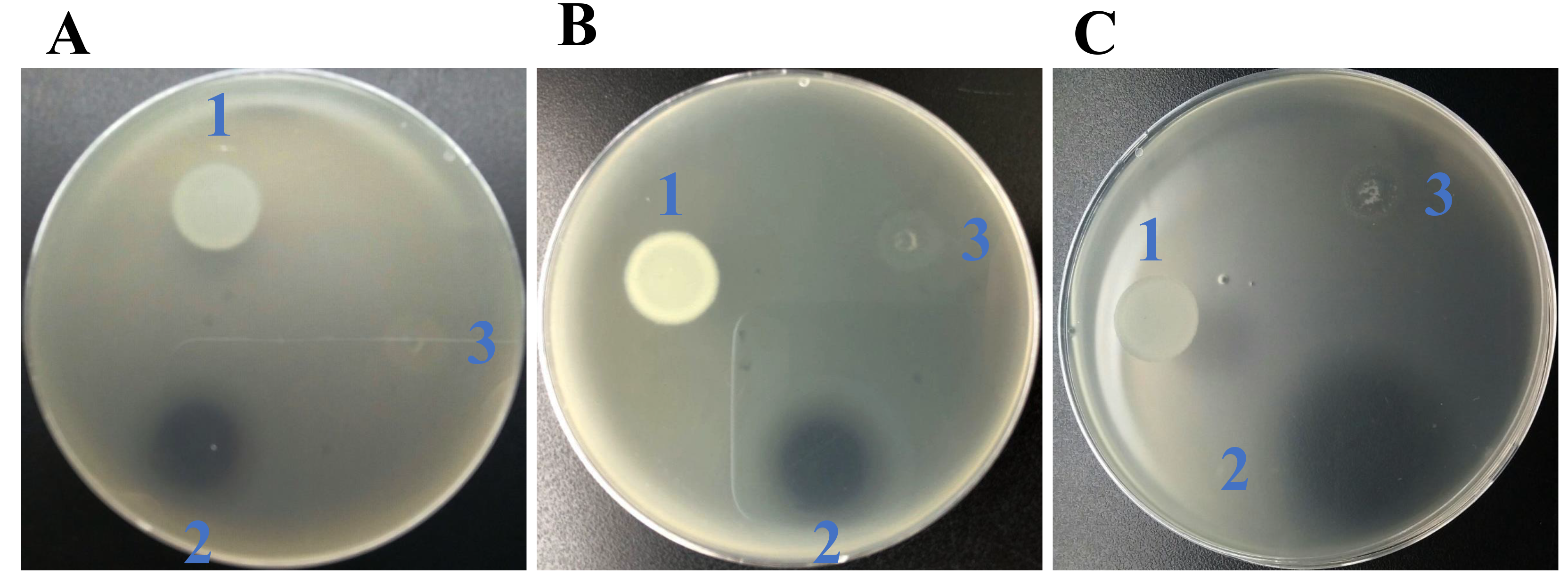
**Supplementary material**

Fig. S1. *Bacillus* sp. BR3 antagonism assays on agar plates. Strain DC3000 (A), Z3-3 (B), or 2P24 (C) was grown in LB at 28 ℃ overnight and mixed with LB agar (ca. 50 ℃) to pour into the Petri dish, respectively. 5 μL of BR3 culture (1), the extract BR3 (32 μg ml^-1^) (2), and kanamycin (50 μg ml^-1^) (3) were spotted on the plates. The plates were inoculated at 28 ℃ for 36 h. There independent experiments were performed; a representative result is shown.
